# Higher Protein Intake Is Not Associated with Decreased Kidney Function in Pre-Diabetic Older Adults Following a One-Year Intervention—A Preview Sub-Study

**DOI:** 10.3390/nu10010054

**Published:** 2018-01-09

**Authors:** Grith Møller, Jens Rikardt Andersen, Christian Ritz, Marta P. Silvestre, Santiago Navas-Carretero, Elli Jalo, Pia Christensen, Elizabeth Simpson, Moira Taylor, J. Alfredo Martinez, Ian Macdonald, Nils Swindell, Kelly A. Mackintosh, Gareth Stratton, Mikael Fogelholm, Thomas M. Larsen, Sally D. Poppitt, Lars O. Dragsted, Anne Raben

**Affiliations:** 1 Department of Nutrition, Exercise and Sports, Faculty of Science, University of Copenhagen, 1958 Copenhagen, Denmark; jra@nexs.ku.dk (J.R.A.); ritz@nexs.ku.dk (C.R.); piach@nexs.ku.dk (P.C.); tml@nexs.ku.dk (T.M.L.); ldra@nexs.ku.dk (L.O.D.); ara@nexs.ku.dk (A.R.); 2 Human Nutrition Unit, School of Biological Sciences, University of Auckland, Auckland 1010, New Zealand; m.silvestre@auckland.ac.nz (M.P.S.); s.poppitt@auckland.ac.nz (S.D.P.); 3 Centre for Nutrition Research, University of Navarra, Calle Lrunlrrea 1, 31008 Pamplona, Navarra, Spain; snavas@unav.es (S.N.-C.); jalfmtz@unav.es (J.A.M.); 4 CIBERobn, Instituto de Salud Carlos III, C/Monforte de Lemos 3–5, 28029 Madrid, Spain; 5 Department of Food and Environmental Sciences, University of Helsinki, 00014 Helsinki, Finland; elli.jalo@helsinki.fi (E.J.); mikael.fogelholm@helsinki.fi (M.F.); 6 School of Life Sciences, Faculty of Medicine and Health Sciences, University of Nottingham, University Park, Nottingham NG7 2RD, UK; Liz.Simpson@nottingham.ac.uk (E.S.); Moira.Taylor@nottingham.ac.uk (M.T.); Ian.Macdonald@nottingham.ac.uk (I.M.); 7 Institute IMDEA Food, Crta. De Canto Blanco 8, 28029 Madrid, Spain; 8 School of Sport and Exercise Sciences, A-STEM Research Centre, Swansea University, Singleton Park SA2 8PP, UK; n.j.swindell.835228@swansea.ac.uk (N.S.); k.mackintosh@swansea.ac.uk (K.A.M.); G.Stratton@swansea.ac.uk (G.S.)

**Keywords:** pre-diabetes, dietary protein, creatinine clearance, glomerular filtration rate, albumin, urea

## Abstract

Concerns about detrimental renal effects of a high-protein intake have been raised due to an induced glomerular hyperfiltration, since this may accelerate the progression of kidney disease. The aim of this sub-study was to assess the effect of a higher intake of protein on kidney function in pre-diabetic men and women, aged 55 years and older. Analyses were based on baseline and one-year data in a sub-group of 310 participants included in the PREVIEW project (PREVention of diabetes through lifestyle Intervention and population studies in Europe and around the World). Protein intake was estimated from four-day dietary records and 24-hour urinary urea excretion. We used linear regression to assess the association between protein intake after one year of intervention and kidney function markers: creatinine clearance, estimated glomerular filtration rate (eGFR), urinary albumin/creatinine ratio (ACR), urinary urea/creatinine ratio (UCR), serum creatinine, and serum urea before and after adjustments for potential confounders. A higher protein intake was associated with a significant increase in UCR (*p* = 0.03) and serum urea (*p* = 0.05) after one year. There were no associations between increased protein intake and creatinine clearance, eGFR, ACR, or serum creatinine. We found no indication of impaired kidney function after one year with a higher protein intake in pre-diabetic older adults.

## 1. Introduction

Prevalence of type 2 diabetes (T2D) is increasing worldwide, imposing a growing burden on individuals, society, and health-care costs [1]. Risk of T2D is determined by an interplay of genetic and metabolic disorders, but overweight and obesity are the predominant risk factors [2]. Results from the US Diabetes Prevention Program, the Finnish Diabetes Prevention Study, and the Chinese Da Qing Study demonstrate that a modest weight loss induced by change of diet and increased physical activity can delay or even prevent the onset of T2D [3,4,5]. Indeed, a weight loss of just 5–10% of body weight leads to positive health benefits [6]. Protein is highly satiating and a high-protein diet has been recommended for the treatment of obesity [7]. Results from both intervention and epidemiological studies have shown that diets rich in protein can result in greater weight loss [8,9,10,11]. However, untoward effects of a high protein intake on kidney function have also been suggested. Evidence of adverse effects of a high-protein diet is inconclusive [12,13,14,15,16].

Concerns regarding adverse renal effects of a high-protein diet (i.e., a protein content of more than 25% of energy consumed (E%) or more than 2 g protein/kg body weight) are related to glomerular hyperfiltration and hypertensive effects [17]. Individuals with obesity-related conditions, such as the metabolic syndrome, pre-diabetes, and T2D, are potentially more susceptible to hypertension than their healthy counterparts [18]. In addition, cross-sectional and longitudinal studies have shown that glomerular filtration rate (GFR) declines with age (decline of approximately 10 mL/min/1.73 m^2^ per decade after the age of 30), making the older population more susceptible to hypertension [19]. The risk of hypertension is exacerbated by a variety of other factors, such as an increase in dietary sodium from a greater intake of animal protein in processed foods. Sodium is used to enhance taste and preserve processed meats, and this can be associated with an increased risk of nephrolithiasis (kidney stones) [7,17,20]. There is also some concern that these changes in protein consumption could lead to an increase in the incidence rate of chronic kidney disease in the general population [17]. Nephropathy is a well-known complication of T2D, and it is relevant to consider this in a vulnerable population.

To the best of our knowledge, no studies have previously investigated the effect of a higher protein intake on the change in kidney function in pre-diabetic overweight or obese individuals who have undergone a period of weight loss and subsequent weight maintenance. The aim of this sub-study of the PREVIEW diabetes prevention study was to investigate whether a higher protein intake during weight loss had an effect on kidney function in a subgroup of older overweight or obese pre-diabetic adults [21]. We hypothesized that a higher protein intake may be associated with a decrease in kidney function, measured by a change in creatinine clearance, after one year of the intervention.

## 2. Materials and Methods

### 2.1. Study Design and Population

This sub-study is part of a 3-year large multi-center randomized controlled trial "PREVIEW" (PREVention of diabetes through lifestyle intervention and population studies in Europe and around the World). The main aim of PREVIEW is to investigate the effects of a high-protein, low-glycemic index (GI) diet versus a moderate protein, medium-GI diet and high versus low intensity physical activity programs (4 intervention arms) in overweight and obese pre-diabetic adults and children in order to find the most efficient lifestyle program in the prevention of T2D. The methodology of PREVIEW has been described elsewhere [21]. In brief, the 3-year intervention consists of two phases: a two-month period of rapid weight reduction achieved using a commercial low-energy diet (LED, about 3.4 MJ/daily), followed by a 34-month randomized lifestyle intervention phase for weight loss maintenance. Clinical investigation days (CIDs) were carried out throughout the intervention, from CID1 (baseline) to CID7 (end of trial). As the PREVIEW intervention study is ongoing and has not been unblinded, the present sub-study treated data as a single cohort. Data from baseline (CID1) and after one year of intervention (CID4) were included in the present sub-study.

Participants aged 55 years or older were included. Selection of participants was performed to get a representative sample from the four intervention arms. We selected the first 110 participants from the subject-ID list from the University of Copenhagen (UCPH, Copenhagen, Denmark) and the first 50 participants from each of the subject-ID lists from the University of Helsinki (HEL, Helsinki, Finland), the University of Auckland (UOA, Auckland, New Zealand), the University of Navarra (UNAV, Pamplona, Spain), and the University of Nottingham (UNOTT, Nottingham, UK). The total number of participants was 310.

### 2.2. Outcome Measures

Kidney function was measured by 24 h creatinine clearance, where creatinine clearance (mL min^−1^) = urinary creatinine concentration × 24 h volume/plasma creatinine concentration × 1440. The estimated glomerular filtration rate (eGFR) was calculated using the Chronic Kidney Disease Epidemiology Collaboration creatinine formula (CKD-EPI_Crea_ formula) [22]. In addition, the urinary albumin/creatinine ratio (ACR), the urinary urea/creatinine ratio (UCR), and the serum urea were measured.

### 2.3. Diet

The two-month weight reduction period included a commercial LED (Cambridge Weight Plan^®^ Ltd., Corby, UK), with a requirement to lose ≥8% of initial body weight in order to continue to the weight maintenance phase. The LED consisted of meal replacements with an energy content of 3.4 MJ/day (810 kcal/day), and a macronutrient composition of 45–50 E% from carbohydrate, 35–40 E% from protein, and 15–20 E% from fat. In addition, an intake of 200 g of tomatoes, 125 g of cucumber, and 100 g of lettuce per day as well as sugar-free beverages providing 0–25 kJ/day were allowed as part of the diet.

Participants who achieved ≥8% body weight loss during the LED period were allocated using block randomization to one of the four study arms for the 34-month weight maintenance phase with the aim of preventing the development of T2D. The intervention had a 2 × 2 factorial design and consisted of two different diets and two different physical activity programs: The two diets were as follows: a Moderate Protein (MP) diet—15 E% protein, 55 E% carbohydrates, and a moderate dietary glycemic index (GI) (≥56)—and a High Protein (HP) diet—25 E% protein, 45 E% carbohydrates, and a lower GI (≤50). Both diets provided a moderate fat intake of 30 E%. The two physical activity groups were defined as follows: one group engaged in high-intensity physical activity for 75 min/week and the other engaged in moderate-intensity physical activity for 150 min/week. Both regimens aimed at an exercise energy expenditure of >4200 kJ/week.

Dietary intake was assessed from an average of four-day food records at CID1 and after one year (CID4). The dietary data were entered and analyzed for energy and protein content using a computerized version of national food composition tables; UCPH, Dankost version 3000 (Dankost, Copenhagen, DK), HEL, AivoDiet (Aivo Finland Oy, Turku, Finland), UNOTT, Nutritics edition v4.2 (Nutritics, Dublin, Ireland) UNAV, Dial (Alceingieria, Madrid, Spain), UOA, Foodworks 8 (Xyris, Brisbane, Australia). Individuals who had an implausibly high (>16,800 kJ) or low (<4200 kJ) daily energy intake were excluded from the study.

Twenty-four-hour urinary urea excretion was used as a biomarker of protein intake and calculated using the formula 24 h urinary urea (mmol/day) × 0.22 + 12.5 g protein/day [23]. In the analyses, protein intake was calculated as g protein/kg body weight/day.

### 2.4. Blood Pressure

Blood pressure was measured at CID1 and CID4 with a calibrated electronic sphygmomanometer. Blood pressure was measured as follows: UCPH, A&D Medical UA-787 Plus (A&D Medical, Tokyo, Japan), HEL, OMRON M2 HEM-7119-E (OMRON Healthcare Co Ltd, Kyoto, Japan), UNAV, Omron M6-Comfort (Omron Healthcare Europe BV, Barcelona, Spain), UNOTT, GE Medical Dash 3000 (GE Healthcare, Freiburg, Germany), UOA, GE (Dynamap V100 (GE Healthcare, Auckland, New Zealand)). Participants were sitting, and measures were taken in the right arm after 5–10 min of rest. Blood pressure was measured three consecutive times with 1 min of rest between each measurement. The average of the three measurements was registered.

### 2.5. Body Composition

Measurements of body composition were performed at CID1 and CID4 by different methods at the intervention sites: UCPH, iDXA, software v.15, (GE-Lunar, Madison, WI, USA), HEL, InBody720 Body Composition Analyzer by Biospace Co., Ltd, manufacturing year 2004; UNAV, TANITA BC-420MA, TANITA corp, UNOTT, GE-Lunar prodigy with eCORE 2005 software v. 9.30.044), UOA, iDXA, software v.15, GE-Lunar.

### 2.6. Physical Activity

Accelerometer-derived data were used to adjust for the possible effect of physical activity on renal perfusion. Participants wore an ActiSleep+ (ActiGraph LLC, Pensacola, FL, USA) accelerometer attached to an elastic waist belt worn over the right mid-axillary line 24 h/day for seven consecutive days, at CID1 and CID4, removing it only for water-based activities. The primary output from the ActiSleep+ is an activity count, which represents raw accelerations that have been rescaled and filtered. Activity counts were collected at 100 Hz and aggregated to 60 epochs. After the removal of nocturnal sleep episodes, participants were included in the analyses if they wore the monitor for ≥10 h on ≥4 days including at least one weekend day. Mean activity counts during valid wear time (counts·min^−1^; CPM) have been shown to correlate well with total activity energy expenditure measured with the doubly labeled water technique and were used as an indicator of total PA volume. Troiano cut points [24] were used to determine time (min·day^−1^) spent at different intensity categories (sedentary: <100 counts per minute (CPM); moderate: 100–2020 CPM; vigorous: 2020–5999 CPM). The duration for moderate-to-vigorous intensity activities were summed to obtain moderate-to-vigorous physical activity (MVPA).

### 2.7. Laboratory Analysis

To decrease inter-laboratory variation in calibration and analysis of the kidney markers, all serum and urine samples were analyzed in the central laboratory of University of Copenhagen.

Venous blood samples were drawn from the antecubital vein at CID1 and CID4 after an overnight fast. Serum was separated from blood cells by 1500 G centrifugation for 10 min, after the sample had been allowed to clot for 15 min and then stored at −80 °C. Urinary concentration of urea, creatinine, and albumin was measured in 24 h-urine collections at CID1 and after one year (CID4). Urine collection was initiated after the first voiding on the collection day at 8:00 a.m. and terminated after the following day’s first voiding at 8:00 a.m. After a weighing and assessment of density, urine was stored at −80 °C prior to further analysis. All serum and urine samples were determined with a HORIBA ABX Pentra 400 analyzer (HORIBA, Montpellier, France).

A standard oral glucose tolerance test (75 g of glucose, dissolved in 300 mL of water) was completed to assess glycemic status. Fasting (0 h) and 2 h blood samples were collected. The blood samples were drawn from the antecubital vein. Serum and whole blood samples were initially stored at −80 °C at the individual intervention sites, after which they were transported to a laboratory in Finland for central batch analyses (National Institute for Health and Welfare, Helsinki, Finland). Laboratory analyses of glucose were performed on an Architect ci8200 integrated system (Abbott Laboratories, Abbott Park, IL, USA).

### 2.8. Statistical Analysis

All of the statistical analyses were performed using R (v.3.3.0, R Core Team, Vienna, Austria) [25]. Only CID1 and CID4 data were included in the complete-case analyses.

Analysis of covariance was used to investigate the association between changes in protein intake (estimated from 24 h urinary urea excretion and four-day dietary records) and changes in each of the kidney health risk markers. Unadjusted analyses as well as analyses adjusted for potential confounders, including age, gender, MVPA, and study center, the latter of which was adjusted as a linear mixed model, since study center was included as a random effect, were conducted. In addition, urea excretion was analyzed by comparing changes among participants in tertiles of protein intake, estimated from 24 h urinary urea excretion. As weight loss and improvement of glycemic control could in themselves be responsible for changes in kidney function, additional analyses including adjustment for change in body weight after one year and subgroup analyses excluding participants achieving improved glycemic control were also carried out (Supplemental Material). These associations were also investigated cross-sectionally for each visit separately (Supplemental Material).

Model checking of assumptions of variance homogeneity and normality were carried out by means of residual and normal probability plots. Self-reported protein intake was correlated to 24 h urinary urea excretion using the Pearson correlation test. A *p*-value < 0.05 was considered statistically significant.

### 2.9. Research Ethics

All subjects gave their informed consent for inclusion before they participated in the study. The study was conducted in accordance with the Declaration of Helsinki, and the protocol was approved by the Ethics Committee of Municipality Region in Denmark 07-03-2013 (H-1-2013-052) and the local ethical committees in the respective countries.

## 3. Results

One participant was excluded due to missing data. Thus, a total of 309 overweight or obese pre-diabetic individuals were included in the complete-case analyses. Descriptive statistics for participants at baseline and after one year are shown in Table 1. There was a significant improvement in all measures, including body weight, fasting plasma glucose (FPG), 2 h plasma glucose, body mass index (BMI), body composition (fat free mass, FFM and fat mass, FM), blood pressure, and activity level parameters after one year (all *p*-values < 0.05). Protein intake in g/day decreased, but protein intake in g/kg body weight/day and estimated protein intake in g/kg body weight/day from urea excretion increased after one year. On average, for all participants kidney function had slightly decreased by CID4 (Table 1).

Change in creatinine clearance, UCR, and serum urea was positively associated with change in estimated protein intake calculated from the urea excretion (*p* < 0.0001, Table 2), both with and without adjustments. There was also a positive association between change in eGFR and change in estimated protein intake calculated from urea excretion (*p* < 0.05, Table 2), but only after adjustments. Change in serum creatinine was inversely associated with change in estimated protein intake calculated from the urea excretion (*p* < 0.05, Table 2), but only after adjustments. No associations were found between change in ACR and change in estimated protein intake calculated from the urea excretion. In addition, change in UCR and serum-urea was positively associated with both change in self-reported protein intake (*p* < 0.05, Table S1) and change in urea excretion (*p* < 0.0001, Table S2), both with and without adjustments. In addition, change in creatinine clearance was positively associated with change in urea excretion (*p* < 0.0001, Table S2). No associations were found between change in self-reported protein and creatinine clearance, eGFR, ACR, or serum creatinine (Table S1) or between change in urea excretion and change in eGFR, ACR, or serum creatinine, respectively (Table S2). Tables S3 and S4 provide data adjusted for body weight, but this standardization did not change the results. In Tables S5 and S6, individuals who improved their glycemic control were excluded from the analyses, but again this did not change the results. Baseline urea excretion (mmol/day) in urine and self-reported total protein intake (g/kg/day) were moderately, positively correlated (r = 0.37, *p* < 0.0001).

When protein intake was estimated from the urea excretion (based on mean values from the tertiles), participants with the highest estimated protein intake (upper tertile) ingested 1.6 (SD 0.4) g protein/kg body weight/day compared to 1.0 (SD 0.6) g protein/kg body weight/day among participants with the lowest intake (*p* < 0.0001). Participants in the middle tertile ingested 1.3 (SD 0.4) g protein/kg body weight/day.

Table 3 shows that there is no indication that increasing protein intake causes detrimental effects on the kidney function, independent of the baseline intake of protein at CID1 (low, moderate, or high intake). There is even an indication (*p* = 0.056) of an improvement in kidney function with increasing protein intake in individuals already ingesting a large amount of protein. Within each tertile, changes in UCR and serum urea do not show a systematic association, as those with a moderate protein intake seem to have the highest increase after further increasing their protein intake. For the remaining markers of kidney function, there were no differences in rates of change across the three tertiles (all *p* > 0.05).

In detail, this means that, for each 1 g/kg/day increase in protein intake, ΔUCR increased significantly more in the moderate intake group than in the low and high intake groups (a difference in slope of −18.35 ± 6.01 g/kg/day, *p* < 0.01, and −12.70 ± 5.49 g/kg/day, respectively, *p* < 0.05, Table 3). Likewise, for each 1 g/kg/day increase in protein intake, ΔS-Urea was higher in the moderate intake group than in the low and high intake groups (a difference in slope of −1.43 ± 0.42 mmol/L/g/kg/day, *p* < 0.001, and −0.93 ± 0.39 mmol/L/g/kg/day, *p* < 0.05).

Tables S3 and S4 provide data adjusted for body weight, but this standardization did not change the results. In Tables S5 and S6, individuals who improved their glycemic control were excluded from the analyses, but again this did not change the results. Tables S7–S10 show the cross-sectional analyses at inclusion and after one year, and this did not change outcomes either.

## 4. Discussion

The main finding of the present study was that a higher protein intake (>1.6 g/kg/day) was not associated with a decreased kidney function after one year of intervention of a weight-loss, weight-maintenance program in pre-diabetic older adults. As expected though, individuals with a high self-reported protein intake had a significantly increased urea-to-creatinine ratio as well as an increased concentration of urea in urine and serum. When we divided the participants into tertile groups, we could not explain why an increase in UCR and serum-urea was only present in the group with a moderate protein intake, unchanged values probably represent adaption.

High-protein diets have long been recognized as a potential modulator of kidney function due to an acute increase in renal plasma flow and GFR [17]. This has raised concerns that ingestion of high-protein diets may increase glomerular pressure and hyperfiltration, which may lead to a progressive loss of kidney function over the long term [17,26]. In contrast, Bankir et al. [27] has stated that an increase in GFR is likely to be a normal adaptation of the kidney to an increased protein intake, with the consequence being a higher urinary urea concentration. The most plausible cause for this may be that urea induces osmotic diuresis [28]. However, evidence to support the hypothesis that prolonged glomerular hyperfiltration leads to kidney damage remains equivocal [29], especially in high risk groups like patients with T2D or insulin resistance.

In healthy individuals, an increase in protein intake up to a maximum of 30 E% has not been shown to adversely affect kidney function [30,31]. The present study in pre-diabetic older adults resulted in the same finding, which also agreed well with findings from a randomized controlled trial among 68 individuals with abdominal obesity [26]. In that investigation, they compared high protein (24 E%)/low carbohydrate (4 E%) and high protein (30 E%)/very low carbohydrate (61 E%) diets for one year and showed that, regardless of diet, there was no change in serum creatinine levels, eGFR, or urinary albumin excretion [26]. In the National Health and Nutrition Examination Survey (NHANES) conducted in 2007–2010 with 11,111 healthy adults, Berryman et al. [12] also found no significant associations between total protein intake and estimated GFR. Similarly, intervention studies with a duration up to one year by Li et al. [32] and Skov et al. [7] showed that protein-enriched meal replacements or high-protein diets did not have adverse effects on kidney function among obese individuals. In a two-year study by Friedman et al. [29] involving 307 obese individuals, a low-carbohydrate, high-protein diet aiming at weight loss, compared with a standard low-fat weight-loss diet, was also not associated with any increase in creatinine clearance, urinary albumin excretion, urinary volume, or serum urea. It is also noteworthy that weight loss and improvement in glycemic control did not affect the kidney function in the present study (Supplemental Material).

In contrast, a recent systematic review and meta-analysis, including a total of 30 randomized controlled trials with varying definitions of high and low protein diets found that higher protein diets were associated with increased GFR and serum urea among healthy individuals or individuals with T2D, but there was no indication of disease or functional problems—only an adaptation [33]. Likewise, in a sub-study of the OmniHeart Trial, in a randomized 3-period, 6-week crossover study, a protein-rich diet increased GFR compared to diets rich in carbohydrate and unsaturated fat [15]. Similar to these findings, Frank et al. [16] showed an increase in GFR with a high-protein diet among 24 healthy young men in a randomized, crossover study including a two-diet protocol of 7 days each. The results from these two short-term studies [15,16] mainly reflect physiological and adaptive changes in kidney function to a high protein load. This supports the findings of our current study that increased urea production may lead to osmotic diuresis, but not to kidney damage. Indeed, in accordance with this hypothesis, the diuresis increased significantly in our present study.

In more susceptible groups, such as individuals with T2D without diabetic kidney disease, there was no indication that a high protein intake would lead to kidney damage [34]. For example, a two-year randomized controlled trial, comparing a low-fat, high-protein diet to a low-fat, high-carbohydrate diet in 419 individuals with T2D, showed no differences in kidney function [35]. Other shorter randomized controlled trials (<12 months) in participants with T2D also failed to detect adverse effects of a high-protein diet on kidney function [36,37,38,39].

According to the NHANES survey conducted in 1999–2006 [40] the incidence rate of chronic kidney disease is >40% among adults with a diagnosis of diabetes and 17.7% among adults with pre-diabetes, respectively. Thus, longer-term follow-up studies are required to determine if long-term adherence to a higher-protein diet eventually results in development and progression of kidney disease. In the present analyses, we found no such indication of kidney damage after one year of intervention.

The strength of the present sub-study is the use of both a subjective and objective method to assess protein intake and, and this may have improved the validity of the results [41]. In addition, the inclusion of a large sample size (from five different countries), a longer-term follow-up, and the use of a variety of markers to examine kidney function are positive aspects of this work. Furthermore, we have controlled for possible confounding factors using an objective assessment of physical activity. Nonetheless, several limitations should be noted, including the fact that GFR was estimated, rather than measured by inulin or radio-isotope-labeled filtration markers, which are considered the “gold standard” for measuring GFR [42]. Whilst serum creatinine is more practical to measure clinically, it can be less accurate and may be more difficult to interpret due to day-to-day variations, especially when concentrations are in the normal range in certain subgroups, including obese patients. Additionally, though measured creatinine clearance requires 24 h urine collection and this was undertaken in the present study, the completeness of the urine collection was not assessed.

## 5. Conclusions

In conclusion, in this study of overweight pre-diabetic older adults from five countries, we found no evidence that a higher protein intake was associated with a decrease in kidney function after one year of intervention. Moreover, we found no indication that increasing protein intake caused differential effects on kidney function among adults with the lowest versus the highest baseline protein intake.

## Figures and Tables

**Table 1:** Characteristics of the pre-diabetic participants at baseline and after one year of intervention (*n* = 309).

Variables	Baseline	1 Year	*p*-Value
**Age** (years)	61.4 ± 4.5	-	-
**Males** % (No.)	57.9 (179)	-	-
**Weight** (kg)	94.6 ± 16.9	85.4 ± 15.9	<0.001
**Energy intake** (kJ/day) ^a^	8616 ± 2169	7154 ± 1994	<0.0001
**Protein intake** (g/day) ^a^	90.6 ± 22.9	85.3 ± 27.9	0.005
**Protein intake** (g/kg body weight/day) ^a^	0.98 ± 0.3	1.03 ± 0.4	0.04
**Protein intake** (E%)	18.3 ± 3.4	20.7 ± 4.3	<0.0001
**Calculated protein intake** (g/day (urea excretion)	105.8 ± 35.7	109.6 ± 40.0	0.114
**Calculated protein intake** (g/kg body weight/day (urea excretion)	1.1 ± 0.4	1.3 ± 0.5	<0.0001
**FPG** mmol/L ^b^	6.2 ± 0.58	6.0 ± 0.54	<0.0001
**2h-glucose** mmol/L ^c^	8.2 ± 2.25	6.8 ± 1.76	<0.0001
**Body-mass index** (kg/m^2^)	33.2 ± 4.6	30.0 ± 4.5	<0.001
**Fat Free Mass** (kg) ^d^	54.6 ± 11.5	53.8 ± 11.1	<0.001
**Fat Mass** (kg) ^d^	38.8 ± 11.4	31.8 ± 11.7	<0.001
**Moderate and vigorous physical activity** (CPM) ^e^	23.8 (38.2, 12.3)	28.1 (46.8, 15.5)	<0.001
**Systolic blood pressure** (mmHg)	134.1 ± 15.8	129.8 ± 14.9	<0.001
**Diastolic blood pressure** (mmHg)	78.2 ± 11.7	75.9 ± 10.3	<0.001
Renal characteristics			
**Total urine volume** (mL) ^f^	1972 ± 737.8	2233 ± 804.2	<0.001
**U-Urea excretion** (mmol/day)	424.1 ± 162.4	441.4 ± 183.6	0.114
**Creatinine clearance** (mL/min)	114.1 ± 38.4	108.7 ± 49.9	0.034
**eGFR** (mL/min/1.73 m^2^)	76.3 ± 13.5	77.1 ± 13.3	0.258
**U-Creatinine excretion** (mmol/day)	13.2 ± 5.3	12.3 ± 5.7	0.009
**Urea/Creatinine Ratio (UCR)**	0.69 ± 1.43	0.74 ± 1.43	<0.0001
**S-Creatinine** (µmol/L)	82.8 ± 16.5	81.7 ± 15.6	0.09
**S-Urea** (mmol/L)	5.6 ± 1.4	5.9 ± 1.4	<0.001
**U-Albumin** (mg/day) ^g^	12.5 (21.4, 8.4)	9.5 (18.1, 7.7)	0.469
**Albumin/Creatinine Ratio (ACR)**	0.8 (1.1, 0.5)	0.9 (1.3, 0.7)	0.339

Characteristics are shown as mean ± standard deviation or median and interquartile range in brackets. Abbreviations: CPM: counts per minute; eGFR: estimated glomerular filtration rate; FPG: fasting plasma glucose; 2h-glucose: 2 h plasma glucose; IQR: interquartile range; S-Creatinine: serum creatinine; S-Urea: serum urea; U-Albumin: urine albumin; U-Creatinine excretion: urinary creatinine excretion; U-Urea excretion: urinary urea excretion. a: *n* = 242; b: 1 year (*n* = 246); c: baseline (*n* = 306); 1 year (*n* = 242); d: *n* = 298; e: *n* = 257; f: *n* = 300; g: 16% have a value >1 (baseline); 13% have a value >1 (1-year).

**Table 2:** Associations between change in estimated protein intake calculated from the urea excretion and change in kidney function from baseline to 1 year.

Δ Estimated Protein Intake (g/kg/Day) Calculated from the Urea Excretion						
Variable	*n*	Unadjusted (β ± SE)	*p*-Value	*n*	Adjusted (β ± SE)	*p*-Value
**Δ Creatinine clearance** (mL/min)	294	75.86 ± 4.31	<0.0001	219	72.86 ± 4.94	<0.0001
**Δ** **eGFR** (mL/min/1.73 m^2^)	309	2.06 ± 1.32	0.118	230	3.42 ± 1.56	0.03
**Δ U-Albumin/U-Creatinine ratio (ACR)**	309	−1.12 ± 4.47	0.09	230	0.53 ± 5.24	0.920
**Δ Urea/Creatinine Ratio (UCR)**	309	9.64 ± 2.22	<0.0001	230	13.53 ± 2.60	<0.0001
**Δ S-Creatinine** (µmol/L)	309	−2.14 ± 1.31	0.104	230	−3.59 ± 1.55	0.02
**Δ S-Urea** (mmol/L)	309	0.62 ± 0.16	<0.001	230	0.84 ± 0.19	<0.0001

All values are beta ± SE. Statistical differences between changes is based on analysis of covariance. Beta is the slope coefficient of outcome measures, per 1 unit change in estimated protein intake (g/kg/day) from urea excretion Model Adjusted for age, gender, physical activity (moderate and vigorous), and study site (the University of Copenhagen, the University of Helsinki, the University of Auckland, the University of Navarra, and the University of Nottingham). Abbreviations: eGFR: estimated glomerular filtration rate; S-Creatinine: serum creatinine; S-Urea: serum urea.

**Table 3:** Slope of change in protein intake and change in kidney function (from baseline to 1 year), within each tertile of low, medium, and high protein intake at baseline (CID1). Protein intake was estimated from urinary urea excretion (*n* = 309).

Slope of Δ Protein Intake (g/kg/Day)				
	Low (*n* = 103)	Moderate (*n* = 103)	High (*n* = 103)	*p*-Value
**Δ Creatinine clearance** (mL/min)/g protein /kg/day	59.45 ± 10.23	62.64 ± 8.19	87.59 ± 5.91	0.056
**ΔeGFR** (mL/min/1.73 m^2^/g protein/kg/day)	2.56 ± 2.48	5.09 ± 2.64	−0.12 ± 1.99	0.626
**Δ Albumin/Creatinine ratio (ACR)**	−5.74 ± 8.40	0.13 ± 8.94	−1.37 ± 6.75	0.434
**Δ Urea/Creatinine Ratio (UCR)**	1.80 ± 4.12	20.16 ± 4.38	7.45 ± 3.31	0.02
**ΔS-Creatinine** (µmol/L/g protein/kg/day)	−2.46 ± 2.48	−4.45 ± 2.63	−0.38 ± 1.99	0.773
**ΔS-Urea** (mmol/L/g protein/kg/day)	−0.02 ± 0.29	1.41 ± 0.31	0.48 ± 0.24	0.006

All values are given as slope coefficient ± SE. *p*-value from F-test for the null hypothesis of no difference across tertiles. The slope coefficient corresponds to the decrease or increase in the change in the marker of kidney function per 1 g/kg/day increase in the protein intake calculated from urea excretion. Abbreviations: eGFR: estimated glomerular filtration rate; S-Creatinine: serum creatinine; S-Urea: serum urea.

## Supplementary Materials

The following are available online at www.mdpi.com/ 2072-6643/10/1/54/s1, Table S1: Associations between change in self-reported protein intake and change in kidney function from baseline to 1 year. Table S2: Associations between change in urea excretion and change in kidney function from baseline to 1 year. Table S3: Associations between change in self-reported protein intake and change in kidney function from baseline to 1 year. Additionally, adjusted for change in body weight. Table S4: Associations between change in urea excretion and change in kidney function from baseline to 1 year. Additionally, adjusted for change in body weight. Table S5: Associations between change in self-reported protein intake and change in kidney function from baseline to 1 year. Individuals who improved their glycemic control are excluded from the statistical analysis. Table S6: Associations between change in urea excretion and change in kidney function from baseline to 1 year. Individuals who improved their glycemic control are excluded from the statistical analysis. Table S7: Associations between baseline self-reported protein intake and baseline markers of the kidney function (CID1). Table S8: Associations between self-reported protein intake and markers of the kidney function after 1 year (CID4). Table S9: Associations between baseline urea excretion and baseline markers of the kidney function (CID1). Table S10: Associations between urea excretion and markers of the kidney function after 1 year (CID4).

## References

[1] Da J., Fernandes R., Ogurtsova K., Linnenkamp U., Guariguata L., Seuring T., Zhang P., Cavan D., Makaroff L.E. (2016). IDF diabetes atlas estimates of 2014 global health expenditures on diabetes. Diabetes Res. Clin. Pract..

[2] Global Report on Diabetes. http://www.who.int/about/licensing/.

[3] The Diabetes Prevention Program (DDP) Research Group (2002). The Diabetes Prevention Program (DDP): Description of lifestyle intervention. Diabetes Care.

[4] Lindström J.O., Louheranta A., Mannelin M., Rastas M., Salminen V., Eriksson J., Uusitupa M., Tuomilehto J. (2003). The Finnish Diabetes Prevention Study (DPS) lifestyle intervention and 3-year results on diet and physical activity. Diabetes Care.

[5] Pan X.R., Li G.W., Hu Y.H., Wang J.X., Yang W.Y., An Z.X., Hu Z.X., Lin J., Xiao J.Z., Cao H.B. (1997). Effects of diet and exercise in preventing NIDDM in people with impaired glucose tolerance: The Da Qing IGT and diabetes study. Diabetes Care.

[6] Goldstein D.J. (1992). Beneficial health effects of modest weight loss. Int. J. Obes. Relat. Metab. Disord..

[7] Skov A.R., Toubro S., Bülow J., Krabbe K., Parving H.H., Astrup A. (1999). Changes in renal function during weight loss induced by high vs low-protein low-fat diets in overweight subjects. Int. J. Obes. Relat. Metab. Disord..

[8] Austin G.L., Ogden L.G., Hill J.O. (2011). Trends in carbohydrate, fat, and protein intakes and association with energy intake in normal-weight, overweight, and obese individuals: 1971–2006. Am. J. Clin. Nutr..

[9] Larsen T.M., Dalskov S.-M., van Baak M., Jebb S.A., Papadaki A., Pfeiffer A.F.H., Martinez J.A., Handjieva-Darlenska T., Kunešová M., Pihlsgård M. (2010). Diets with high or low protein content and glycemic index for weight-loss maintenance. N. Engl. J. Med..

[10] Skov A.R., Toubro S., Rønn B., Holm L., Astrup A. (1999). Randomized trial on protein vs carbohydrate in ad libitum fat reduced diet for the treatment of obesity. Int. J. Obes. Relat. Metab. Disord..

[11] Westerterp-Plantenga M.S., Lejeune M.P.G.M., Nijs I., van Ooijen M., Kovacs E.M.R. (2004). High protein intake sustains weight maintenance after body weight loss in humans. Int. J. Obes. Relat. Metab. Disord..

[12] Berryman C.E., Agarwal S., Lieberman H.R., Fulgoni V.L., Pasiakos S.M. (2016). Diets higher in animal and plant protein are associated with lower adiposity and do not impair kidney function in US adults. Am. J. Clin. Nutr..

[13] Halbesma N., Bakker S.J.L., Jansen D.F., Stolk R.P., De Zeeuw D., De Jong P.E., Gansevoort R.T. (2009). High protein intake associates with cardiovascular events but not with loss of renal function. J. Am. Soc. Nephrol..

[14] Knight E.L., Stampfer M.J., Hankinson S.E., Spiegelman D., Curhan G.C. (2003). The impact of protein intake on renal function decline in women with normal renal function or mild renal insufficiency. Ann. Intern. Med..

[15] Juraschek S.P., Appel L.J., Anderson C.A.M., Miller E.R. (2013). Effect of a high-protein diet on kidney function in healthy adults: results from the omniheart trial. Am. J. Kidney. Dis..

[16] Frank H., Graf J., Amann-Gassner U., Bratke R., Daniel H., Heemann U., Hauner H. (2009). Effect of short-term high-protein compared with normal-protein diets on renal hemodynamics and associated variables in healthy young men. Am. J. Clin. Nutr..

[17] Marckmann P., Osther P., Pedersen A.N., Jespersen B. (2015). High-protein diets and renal health. J. Ren. Nutr..

[18] Leidy H.J., Clifton P.M., Astrup A., Wycherley T.P., Westerterp-Plantenga M.S., Luscombe-Marsh N.D., Woods S.C., Mattes R.D. (2015). The role of protein in weight loss and maintenance. Am. J. Clin. Nutr..

[19] King A.J., Levey A.S. (1993). Dietary protein and renal function. J. Am. Soc. Nephrol..

[20] Brenner B.M., Meyer T.W., Hostetter T.H. (1982). Dietary protein intake and the progressive nature of kidney disease: the role of hemodynamically mediated glomerular injury in the pathogenesis of progressive glomerular sclerosis in aging, renal ablation, and intrinsic renal disease. N. Engl. J. Med..

[21] Fogelholm M., Larsen T., Westerterp-Plantenga M., Macdonald I., Martinez J., Boyadjieva N., Poppitt S., Schlicht W., Stratton G., Sundvall J. (2017). Preview: Prevention of diabetes through lifestyle intervention and population studies in Europe and around the World. Design, methods, and baseline participant description of an adult cohort enrolled into a three-year randomised clinical trial. Nutrients.

[22] Levey A.S., Stevens L.A., Schmid C.H., Zhang Y.L., Castro A.F., Feldman H.I., Kusek J.W., Eggers P., Van Lente F., Greene T. (2009). A new equation to estimate glomerular filtration rate. Ann. Intern. Med..

[23] Souba W.W., Wilmore D.W., Shills M.E., Olson J.A., Shike M., Ross A.C. (1994). Diet and nutrition in the care of the patient with surgery, trauma and sepsis. Modern Nutrition in Health and Disease.

[24] Troiano R.P., Berrigan D., Dodd K.W., Mâsse L.C., Tilert T., Mcdowell M. (2008). Physical activity in the United States measured by accelerometer. Med. Sci. Sports Exerc..

[25] (2016). R: A Language and Environment for Statistical Computing.

[26] Brinkworth G.D., Buckley J.D., Noakes M., Clifton P.M. (2010). Renal function following long-term weight loss in individuals with abdominal obesity on a very-low-carbohydrate diet vs high-carbohydrate diet. J. Am. Diet. Assoc..

[27] Bankir L., Bouby N., Trinh-Trang-Tan M.-M., Ahloulay M., Promeneur D. (1996). Direct and indirect cost of urea excretion. Kidney Int..

[28] Boron W.F., Boulpaep E. (2016). Medical Physiology.

[29] Friedman A.N., Ogden L.G., Foster G.D., Klein S., Stein R., Miller B., Hill J.O., Brill C., Bailer B., Rosenbaum D.R. (2012). Comparative effects of low-carbohydrate high-protein versus low-fat diets on the kidney. Clin. J. Am. Soc. Nephrol..

[30] Dietary Reference Intakes for Energy, Carbohydrate, Fiber, Fat, Fatty Acids, Cholesterol, Protein, and Amino Acids. http://www.nap.edu.

[31] Pedersen A.N., Kondrup J., Børsheim E. (2013). Health effects of protein intake in healthy adults: A systematic literature review. Food Nutr. Res..

[32] Li Z., Treyzon L., Chen S., Yan E., Thames G., Carpenter C.L. (2010). Protein-enriched meal replacements do not adversely affect liver, kidney or bone density: An outpatient randomized controlled trial. Nutr. J..

[33] Schwingshackl L., Hoffmann G., Beck G., Caggiula A., Kusek J. (2014). Comparison of high vs. normal/low protein diets on renal function in subjects without chronic kidney disease: A systematic review and meta-analysis. PLoS ONE.

[34] American Diabetes Association (2017). Lifestyle Management. Diabetes Care.

[35] Krebs J.D., Elley C.R., Parry-Strong A., Lunt H., Drury P.L., Bell D.A., Robinson E., Moyes S.A., Mann J.I. (2012). The Diabetes Excess Weight Loss (DEWL) trial: A randomised controlled trial of high-protein versus high-carbohydrate diets over 2 years in type 2 diabetes. Diabetologia.

[36] Tay J., Thompson C.H., Luscombe-Marsh N.D., Noakes M., Buckley J.D., Wittert G.A., Brinkworth G.D. (2015). Long-term effects of a very low carbohydrate compared with a high carbohydrate diet on renal function in individuals with type 2 diabetes. Medicine (Baltimore).

[37] Larsen R.N., Mann N.J., Maclean E., Shaw J.E. (2011). The effect of high-protein, low-carbohydrate diets in the treatment of type 2 diabetes: A 12 month randomised controlled trial. Diabetologia.

[38] Jesudason D.R., Pedersen E., Clifton P.M. (2013). Weight-loss diets in people with type 2 diabetes and renal disease: A randomized controlled trial of the effect of different dietary protein amounts. Am. J. Clin. Nutr..

[39] Nuttall F.Q., Gannon M.C. (2006). The metabolic response to a high-protein, low-carbohydrate diet in men with type 2 diabetes mellitus. Metabolism.

[40] Plantinga L.C., Crews D.C., Coresh J., Miller E.R., Saran R., Yee J., Hedgeman E., Pavkov M., Eberhardt M.S., Williams D.E. (2010). Prevalence of chronic kidney disease in US adults with undiagnosed diabetes or prediabetes. Clin. J. Am. Soc. Nephrol..

[41] Bingham S.A. (2003). Urine nitrogen as a biomarker for the validation of dietary protein intake. J. Nutr..

[42] Jesudason D.R., Clifton P. (2012). Interpreting different measures of glomerular filtration rate in obesity and weight loss: Pitfalls for the clinician. Int. J. Obes..

