# Supplementary material for: Higher Protein Intake Is Not Associated with Decreased Kidney Function in Pre-Diabetic Older Adults Following a One-Year Intervention—A Preview Sub-Study
Source: Nutrients. 2018 Jan 9;10(1):54. doi: 10.3390/nu10010054 (PMC5793282; doi:10.3390/nu10010054)
Supplement: Supplementary file 1 [file nutrients-10-00054-s001.pdf]

Supplementary Material

Table S1 Associations between change in self-reported protein intake and change in kidney function from baseline to 1 year.

Δ Protein Intake (g/kg/day)						
Variable	**n**	**Unadjusted (ß ± SE)**	***p*-Value**	**n**	**Adjusted (ß ± SE)**	***p*-Value**
**Δ Creatinine clearance** (mL/min)	233	-6.70 ± 9.85	0.497	177	-1.70 ± 10.91	0.876
**Δ eGFR** (mL/min/1.73m^2)	242	0.43 ± 2.14	0.842	183	-0.24 ± 2.55	0.925
**Δ Albumin/Creatinine ratio (ACR)**	242	-2.62 ± 7.13	0.714	183	-1.09 ± 9.52	0.908
**Δ Urea/Creatinine Ratio (UCR)**	242	7.77 ± 3.76	0.04	183	9.93 ± 4.45	0.03
**Δ S-Creatinine** (µmol/L)	242	-1.72 ± 2.13	0.420	183	-1.31 ± 2.51	0.602
**Δ S-Urea** (mmol/L)	242	0.51 ± 0.26	0.05	183	0.62 ± 0.31	0.05

All values are beta ± SE. Statistical differences between changes are based on analysis of covariance.

Beta is the slope coefficient of outcome measures per 1 unit change in protein intake (g/kg/day)

Model Adjusted for age, gender, physical activity (moderate and vigorous), and study site (UCPH, HEL, UOA, UNAV, and UNOTT). Abbreviations: eGFR: estimated glomerular filtration rate; S-Creatinine: serum creatinine; S-Urea: serum urea.

Table S2. Associations between change in urea excretion and change in kidney function from baseline to 1 year

Δ Urea Excretion (mmol/day)						
Variable	**n**	**Unadjusted (ß ± SE)**	***p*-Value**	**n**	**Adjusted (ß ± SE)**	***p*-Value**
**Δ Creatinine clearance** (mL/min)	294	0.22 ± 0.01	<.0001	177	0.21 ± 0.01	<.0001
**Δ eGFR** (mL/min/1.73m^2)	309	0.003 ± 0.004	0.366	183	0.008 ± 0.04	0.07
**Δ Albumin/Creatinine ratio (ACR)**	309	-0.0004 ± 0.02	0.978	183	0.0005 ± 0.02	0.975
**Δ Urea/Creatinine Ratio (UCR)**	309	0.03 ± 0.007	<.0001	183	0.04 ± 0.008	<.0001
**Δ S-Creatinine** (µmol/L)	309	-0.003 ± 0.004	0.408	183	-0.008 ± 0.004	0.08
**Δ S-Urea** (mmol/L)	309	0.002 ± 0.0004	<.0001	183	0.002 ± 0.0005	<.0001

All values are beta ± SE. Statistical differences between changes are based on analysis of covariance.

Beta is the slope coefficient of outcome measures per 1 unit change in urea excretion (mmol/day)

Model Adjusted for age, gender, physical activity (moderate and vigorous), and study site (UCPH, HEL, UOA, UNAV, and UNOTT). Abbreviations: eGFR: estimated glomerular filtration rate; S-Creatinine: serum creatinine; S-Urea: serum urea.

Table S3 Associations between change in self-reported protein intake and change in kidney function from baseline to 1 year. Additionally adjusted for change in body weight.

Δ Protein Intake (g/kg/day)						
Variable	**n**	**Unadjusted (ß ± SE)**	***p*-Value**	**n**	**Adjusted (ß ± SE)**	***p*-Value**
**Δ Creatinine clearance** (mL/min)	233	-6.70 ± 9.85	0.497	193	-2.87 ± 10.12	0.777
**Δ eGFR** (mL/min/1.73m^2)	242	0.43 ± 2.14	0.842	202	-1.13 ± 2.36	0.633
**Δ Albumin/Creatinine ratio (ACR)**	242	-2.62 ± 7.13	0.714	202	-021 ± 8.59	0.981
**Δ Urea/Creatinine Ratio (UCR)**	242	7.77 ± 3.76	0.04	202	6.91 ± 4.29	0.108
**Δ S-Creatinine** (µmol/L)	242	-1.72 ± 2.13	0.420	202	-0.43 ± 2.32	0.852
**Δ S-Urea** (mmol/L)	242	0.51 ± 0.26	0.05	202	0.45 ± 0.29	0.125

All values are beta ± SE. Statistical differences between changes are based on analysis of covariance.

Beta is the slope coefficient of outcome measures per 1 unit change in protein intake (g/kg/day)

Model Adjusted for age, change in body weight from baseline to 1 year follow-up, gender, physical activity (moderate and vigorous), and study site (UCPH, HEL, UOA, UNAV, and UNOTT)

Abbreviations: eGFR: estimated glomerular filtration rate; S-Creatinine: serum creatinine; S-Urea: serum urea.

Table S4. Associations between change in urea excretion and change in kidney function from baseline to 1 year. Additionally adjusted for change in body weight.

Δ Urea Excretion (mmol/day)						
Variable	**n**	**Unadjusted (ß ± SE)**	***p*-Value**	**n**	**Adjusted (ß ± SE)**	***p*-Value**
**Δ Creatinine clearance** (mL/min)	294	0.22 ± 0.01	<.0001	219	0.20 ± 0.01	<.0001
**Δ eGFR** (mL/min/1.73m^2)	309	0.003 ± 0.004	0.366	230	0.007 ± 0.004	0.06
**Δ Albumin/Creatinine ratio (ACR)**	309	-0.0004 ± 0.02	0.978	230	0.004 ± 0.014	0.786
**Δ Urea/Creatinine Ratio (UCR)**	309	0.03 ± 0.007	<.0001	230	0.03 ± 0.007	<.0001
**Δ S-Creatinine** (µmol/L)	309	-0.003 ± 0.004	0.408	230	-0.007 ± 0.004	0.04*
**Δ S-Urea** (mmol/L)	309	0.002 ± 0.0004	<.0001	230	0.002 ± 0.0004	<.0001

All values are beta ± SE. Statistical differences between changes are based on analysis of covariance.

Beta is the slope coefficient of outcome measures per 1 unit change in urea excretion (mmol/day)

Model Adjusted for age, gender, change in body weight from baseline to 1 year follow-up, physical activity (moderate and vigorous), and study site (UCPH, HEL, UOA, UNAV, and UNOTT). Abbreviations: eGFR: estimated glomerular filtration rate; S-Creatinine: serum creatinine; S-Urea: serum urea.

Table S5 Associations between change in self-reported protein intake and change in kidney function from baseline to 1 year. Individuals who improved their glycemic control (n = 191) are excluded from the statistical analysis.

Δ Protein Intake (g/kg/day)			
Variable	**n**	**Unadjusted (ß ± SE)**	***p*-Value**
**Δ Creatinine clearance** (mL/min)	49	6.27 ± 4.97	0.213
**Δ eGFR** (mL/min/1.73m^2)	47	13.24 ± 17.2	0.446
**Δ Albumin/Creatinine ratio (ACR)**	49	-0.85 ± 2.20	0.701
**Δ Urea/Creatinine Ratio (UCR)**	49	14.19 ± 8.65	0.108
**Δ S-Creatinine** (µmol/L)	49	-8.44 ± 4.75	0.08
**Δ S-Urea** (mmol/L)	49	0.62 ± 0.55	0.263

All values are beta ± SE. Statistical differences between changes are based on analysis of covariance.

Beta is the slope coefficient of outcome measures per 1 unit change in protein intake (g/kg/day)

Abbreviations: eGFR: estimated glomerular filtration rate; S-Creatinine: serum creatinine; S-Urea: serum urea.

Table S6. Associations between change in urea excretion and change in kidney function from baseline to 1 year. Individuals who improved their glycemic control (n = 191) are excluded from the statistical analysis.

Δ Urea Excretion (mmol/day)			
Variable	**n**	**Unadjusted (ß ± SE)**	***p*-Value**
**Δ Creatinine clearance** (mL/min)	59	0.17 ± 0.02	<.0001
**Δ eGFR** (mL/min/1.73m^2)	61	0.01 ± 0.009	0.137
**Δ Albumin/Creatinine ratio (ACR)**	61	0.007 ± 0.004	0.076
**Δ Urea/Creatinine Ratio (UCR)**	61	0.03 ± 0.01	0.06
**Δ S-Creatinine** (µmol/L)	61	-0.01 ± 0.008	0.146
**Δ S-Urea** (mmol/L)	61	0.001 ± 0.001	0.147

All values are beta ± SE. Statistical differences between changes are based on analysis of covariance.

Beta is the slope coefficient of outcome measures per 1 unit change in urea excretion (mmol/day)

Abbreviations: eGFR: estimated glomerular filtration rate; S-Creatinine: serum creatinine; S-Urea: serum urea.

Table S7 Associations between baseline self-reported protein intake and baseline markers of the kidney function (CID1).

		Protein Intake (g/kg/day)	
Variable	**n**	**Unadjusted (ß ± SE)**	***p*-Value**
**Creatinine clearance** (mL/min)	262	-6.71 ± 9.02	0.458
**eGFR** (mL/min/1.73m^2)	266	-0.94 ± 3.11	0.764
**U-Albumin/U-Creatinine ratio (ACR)**	266	-26.51 ± 15.88	0.09
**Urea/Creatinine Ratio (UCR)**	266	17.34 ± 4.03	<.0001
**S-Creatinine** (µmol/L)	266	-1.75 ± 3.70	0.636
**S-Urea** (mmol/L)	266	1.32 ± 0.32	<.0001

All values are beta ± SE. Statistical analysis is based on analysis of covariance

Beta is the slope coefficient of outcome measures per 1 unit change in protein intake (g/kg/day)

Model Adjusted for age, gender, physical activity (moderate and vigorous), and study site (UCPH, HEL, UOA, UNAV, and UNOTT). Abbreviations: eGFR: estimated glomerular filtration rate; S-Creatinine: serum creatinine; S-Urea: serum urea.

Table S8 Associations between self-reported protein intake and markers of the kidney function after 1 year (CID4).

		Protein Intake (g/kg/day)	
Variable	**n**	**Unadjusted (ß ± SE)**	***p*-Value**
**Creatinine clearance** (mL/min)	258	5.19 ± 7.69	0.50
**eGFR** (mL/min/1.73m^2)	266	-0.73 ± 2.26	0.75
**U-Albumin/U-Creatinine ratio (ACR)**	266	-31.78 ± 17.91	0.07
**Urea/Creatinine Ratio (UCR)**	266	7.55 ± 3.54	0.03
**S-Creatinine** (µmol/L)	266	-1.70 ± 2.61	0.52
**S-Urea** (mmol/L)	266	0.46 ± 0.25	0.06

All values are beta ± SE. Statistical analysis is based on analysis of covariance.

Beta is the slope coefficient of outcome measures per 1 unit change in protein intake (g/kg/day)

Model Adjusted for age, gender, physical activity (moderate and vigorous), and study site (UCPH, HEL, UOA, UNAV, and UNOTT). Abbreviations: eGFR: estimated glomerular filtration rate; S-Creatinine: serum creatinine; S-Urea: serum urea.

Table S9. Associations between baseline urea excretion and baseline markers of the kidney function (CID1).

		Urea Excretion (mmol/day)	
Variable	**n**	**Unadjusted (ß ± SE)**	***p*-Value**
**Creatinine clearance** (mL/min)	303	0.18 ± 0.01	<.0001
**eGFR** (mL/min/1.73m^2)	309	0.003 ± 0.005	0.559
**U-Albumin/U-Creatinine ratio (ACR)**	309	-0.03 ± 0.02	0.216
**Urea/Creatinine Ratio (UCR)**	309	0.01 ± 0.006	0.04*
**S-Creatinine** (µmol/L)	309	0.02 ± 0.006	<0.01
**S-Urea** (mmol/L)	309	0.002 ± 0.005	<.0001

All values are beta ± SE. Statistical analysis is based on analysis of covariance.

Beta is the slope coefficient of outcome measures per 1 unit change in urea excretion (mmol/day)

Model Adjusted for age, gender, physical activity (moderate and vigorous), and study site (UCPH, HEL, UOA, UNAV, and UNOTT). Abbreviations: eGFR: estimated glomerular filtration rate; S-Creatinine: serum creatinine; S-Urea: serum urea.

Table S10. Associations between urea excretion and markers of the kidney function after 1 year (CID4).

Urea Excretion (mmol/day)			
Variable	**n**	**Unadjusted (ß ± SE)**	***p*-Value**
**Creatinine clearance** (mL/min)	303	0.19 ± 0.009	<.0001
**eGFR** (mL/min/1.73m^2)	309	0.005 ± 0.004	0.207
**U-Albumin/U-Creatinine ratio (ACR)**	309	-0.03 ± 0.03	0.267
**Urea/Creatinine Ratio (UCR)**	309	0.02 ± 0.006	<.0001
**S-Creatinine** (μmol/L)	309	0.02 ± 0.006	0.052
**S-Urea** (mmol/L)	309	0.002 ± 0.005	<.0001

All values are beta ± SE. Statistical analysis is based on analysis of covariance.

Beta is the slope coefficient of outcome measures per 1 unit change in urea excretion (mmol/day)

Model Adjusted for age, gender, physical activity (moderate and vigorous), and study site (UCPH, HEL, UOA, UNAV, and UNOTT). Abbreviations: eGFR: estimated glomerular filtration rate; S-Creatinine: serum creatinine; S-Urea: serum urea.
